# The Impact of an Object’s Surface Material and Preparatory Actions on the Accuracy of Optical Coordinate Measurement

**DOI:** 10.3390/ma18153693

**Published:** 2025-08-06

**Authors:** Danuta Owczarek, Ksenia Ostrowska, Jerzy Sładek, Adam Gąska, Wiktor Harmatys, Krzysztof Tomczyk, Danijela Ignjatović, Marek Sieja

**Affiliations:** 1 Laboratory of Coordinate Metrology, Faculty of Mechanical Engineering, Cracow University of Technology, Jana Pawla II 37, 31-864 Cracow, Poland; danuta.owczarek@pk.edu.pl (D.O.); ksenia.ostrowska@pk.edu.pl (K.O.); jerzy.sladek@pk.edu.pl (J.S.); adam.gaska@pk.edu.pl (A.G.); wiktor.harmatys@pk.edu.pl (W.H.); 2 Faculty of Electrical and Computer Engineering, Cracow University of Technology, Warszawska 24, 31-155 Cracow, Poland; marek.sieja@pk.edu.pl; 3 CosmodromEdu, 8 Allée René Dumont, 67400 Illkirch, France; dani@cosmodromedu.net

**Keywords:** optical coordinate measurement, point cloud, laser triangulation, laser probe, articulated arm, accuracy, metrology

## Abstract

Optical coordinate measurement is a universal technique that aligns with the rapid development of industrial technologies and new materials. Nevertheless, can this technique be consistently effective when applied to the precise measurement of all types of materials? As shown in this article, an analysis of optical measurement systems reveals that some materials cause difficulties during the scanning process. This article details the matting process, resulting, as demonstrated, in lower measurement uncertainty values compared to the pre-matting state, and identifies materials for which applying a matting spray significantly improves the measurement quality. The authors propose a classification of materials into easy-to-scan and hard-to-scan groups, along with specific procedures to improve measurements, especially for the latter. Tests were conducted in an accredited Laboratory of Coordinate Metrology using an articulated arm with a laser probe. Measured objects included spheres made of ceramic, tungsten carbide (including a matte finish), aluminum oxide, titanium nitride-coated steel, and photopolymer resin, with reference diameters established by a high-precision Leitz PMM 12106 coordinate measuring machine. Diameters were determined from point clouds obtained via optical measurements using the best-fit method, both before and after matting. Color measurements using a spectrocolorimeter supplemented this study to assess the effect of matting on surface color. The results revealed correlations between the material type and measurement accuracy.

## 1. Introduction

Coordinate optical systems are universal solutions enabling accurate measurement, including in measurement tasks that are impossible to perform using classic contact methods. They are widely used in manufacturing and the aerospace industry for precise measurement and quality control. The most common technologies on the market use triangulation and time-of-flight (TOF), i.e., laser-based measurement systems, as well as photogrammetry or fringe projection [1]. Examples of their applications are given, for instance, in [2,3,4,5]. According to [6], the “Optical Coordinate Measuring Machine Market is expected to grow from USD 1.72 Billion in 2025 to USD 2.48 Billion With CAGR to be around 4.13% during 2034”.

Currently, measurement solutions using both contact and non-contact techniques in a complex form, such as multisensory machines, are common on the market. In these machines, contact measurements are used mainly in narrow tolerance zones [7], while optical measurements significantly accelerate the measurement process [8]. The advantage of multisensory systems lies in the fact that, by offering various measurement possibilities, they become an even more universal tool. Due to a complex shape or the type of material used in a coating, not all objects can be measured by contact. This limitation is especially observed in biomedical applications, for instance when measuring soft materials such as tissues, or when working with modern materials.

The operating principles of optical systems fundamentally differ from those of contact systems, necessitating their separate consideration. The issue of factors causing the largest errors in optical measurements, alongside the estimation of measurement uncertainty, has been extensively studied over the years by leading scientific and research centers as well as National Metrology Institutes (NMIs), including the National Institute of Standards and Technology (NIST) in the United States, Physikalisch-Technische Bundesanstalt (PTB) in Germany, and the National Physical Laboratory (NPL) in the United Kingdom [9,10,11]. In the context of optical measurement uncertainty, researchers consider factors such as illumination, air contamination, and vibrations [9], as well as illumination conditions, objective magnification, measuring window size, and autofocus capabilities [10]. Some studies also focus on the impact of calibration standards on machine accuracy, including the appropriate selection of calibration standard materials [8]. The work presented in [12] highlights the interaction between 3D optical scanners and objects composed of different materials and colors. Furthermore, ref. [13] identifies a systematic noise error dependent on the color of the object, demonstrating that under identical illumination some colors exhibit pronounced noise errors, whereas others show minimal or no systematic errors.

**Figure 1:**
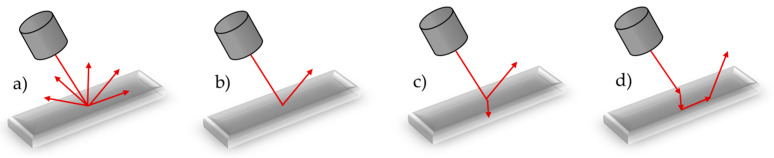
Diagram illustrating the reflection of laser light on various surface types: (**a**) diffuse object, (**b**) specular object, (**c**) transparent object, and (**d**) translucent object [15,16].

The accuracy of optical measurement systems is significantly influenced by various environmental factors, as well as the surface characteristics of the measured object, including reflectivity (which leads to undesired light reflection), transparency (resulting in the penetration of light into the object), and the object’s color (which affects measurement performance, particularly in structured light techniques) [14]. It is crucial to distinguish whether the object being measured is (a) diffuse, (b) specular, (c) transparent, or (d) translucent (Figure 1) [15,16].

Work is currently underway to counteract the unfavorable impact of reflectivity on the measurement result [17]. Scientists propose, among others, the following solutions: a 3D scanning technique for reflective metal surfaces based on an HDR-like image generated via a pseudo-exposure image fusion method [18]; an infrared system for the 3D scanning of metallic surfaces [19]; or the technique proposed by Speck et al., ‘shape from heating’, as an appropriate method to capture non-cooperative surfaces [20,21]. In [20], the experimental implementation of a thermal 3D sensor based on sequential fringe projection is presented.

To mitigate undesirable effects, specialized 3D scanning sprays are commonly used. These sprays reduce surface gloss, create a uniform coating, and enhance contrast, thereby improving measurement accuracy—particularly for objects that would be challenging or impossible to measure without the matting process. Research on matting layers has been conducted [22], and development of novel solutions continues [23]. Nonetheless, as demonstrated in [15], while matting enhances measurement accuracy or enables the measurement itself, it also introduces interference with the surface layer, with a thickness ranging approximately from 1 to 30 µm. The range on the level of a few micrometers is comparable to the accuracy achievable by coordinate measuring machines.

This article emphasizes the importance of deliberate planning in the measurement process using coordinate optical measurement techniques. It presents observed regularities in measurements of objects, both with and without the application of a matting product, focusing on the accuracy of dimensional reproduction—the diameters of spheres fabricated from ceramics, tungsten carbide, matte-finished tungsten carbide, aluminum oxide, titanium nitride-coated steel, and photopolymer resin—relative to reference values. Measurements were performed using the Romer Absolute Arm 7320RI system from Hexagon AB (Publ) (Stockholm, Sweden) equipped with a Scanner RS2 laser head, alongside the Leitz PMM 12106 coordinate measuring machine from Hexagon AB (Publ) (Stockholm, Sweden). In the context of this article’s novelty, Section 3 identifies the materials for which the application of a matting spray is particularly necessary. The authors further propose a classification of materials into easy-to-scan and hard-to-scan categories, accompanied by procedures aimed at improving measurement accuracy, particularly for the latter group. This article also includes new content referring to measurement uncertainty, which—according to the results—decreases when a matting agent is used. Furthermore, this article includes a colorimetric analysis conducted during the matting process using a spectrocolorimeter, aimed at evaluating the effectiveness of surface coverage by the matting agent. To the best of the authors’ knowledge, such analyses have not been previously undertaken. The results provide important information about the completeness of matting layer coverage, which is highly significant when measuring objects classified as hard to scan. The conducted studies and analyses provide valuable knowledge both in the context of the materials used and the metrological processes involved. This article concludes with a comprehensive summary of the findings and corresponding conclusions.

## 2. Materials and Methods

The tests were performed in the accredited Laboratory of Coordinate Metrology at the Cracow University of Technology in a room with a constant temperature of 20 ± 0.5 °C. The laboratory is equipped with a continuous temperature monitoring system, enabling the operator to check the temperature values during the measurement process. Prior to measurement, precautions were taken to ensure that any objects previously outside the laboratory were thermally stabilized, cleaned of contaminants, and mounted on the measuring table in a manner that allows unrestricted movement.

**Figure 2:**
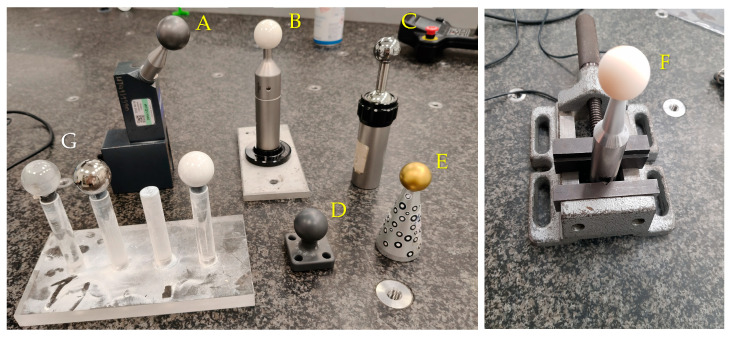
Reference spheres made of various materials used in tests: matte-finished tungsten carbide (A), ceramic (B), tungsten carbide (C), photopolymer resin (D), titanium nitride-coated steel (E), and aluminum oxide (F); a crystal ball used for the preliminary test (G) and the sphere mounting.

Standard spheres were used, representing a diversity of materials used in tests (Figure 2):

− Ceramic sphere ϕ30.00050 mm;
− Matte-finished tungsten carbide sphere ϕ29.99723 mm;
− Tungsten carbide sphere ϕ25.00039 mm;
− Aluminum oxide sphere ϕ24.99013 mm;
− Titanium nitride-coated steel sphere ϕ24.98043 mm;
− Photopolymer resin sphere ϕ29.99300 mm.

The reference standard set was intentionally selected to represent a broad spectrum of surface characteristics while also incorporating materials commonly used in the production of calibration standards.

**Figure 3:**
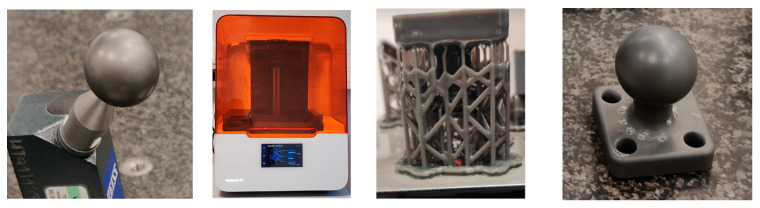
Printing a replica of the reference sphere using SLA technology. From left to right: calibration sphere, the printing machine, the model with supports, and the printed model.

One of the spheres was made using Stereolithography (SLA) additive printing technology on a Formlabs Form 3B + 3D printer from Formlabs Inc. (Somerville, MA, USA). The material used was Tough 2000 photopolymer resin from Formlabs, which is characterized by its high tensile strength and resistance to damage: ultimate tensile strength, 46 MPa; tensile modulus, 2.2 GPa; elongation, 48%; and flexural modulus, 1.9 GPa [24]. The sphere was processed and placed in the FormWash washer. This process was intended to clean the elements of excess resin. After cleaning, the model was placed in a FormCure UV chamber (Formlabs), where the resin hardened for an hour at 70 °C. The printed sphere is the beginning of the concept described by Harmatys et al. in [25] covering the idea of printing dedicated patterns in SLA technology (Figure 3). The use of additive printing technology in metrology is only one of the wide applications offered by this technology, e.g., [26].

Preliminary tests were conducted to assess the feasibility of acquiring measurement data using a laser probe on transparent objects. In the initial stage, a fully transparent sphere was measured (Figure 2G), followed by a semi-transparent reference standard made of photopolymer resin. The measurement procedure for preliminary tests involved a single pass of the laser probe, analogous to the methodology described below, during which point cloud data were collected. The objective of these preliminary tests was limited to verifying the possibility of point acquisition on such surfaces, without performing an accuracy analysis of the resulting scan.

**Figure 4:**
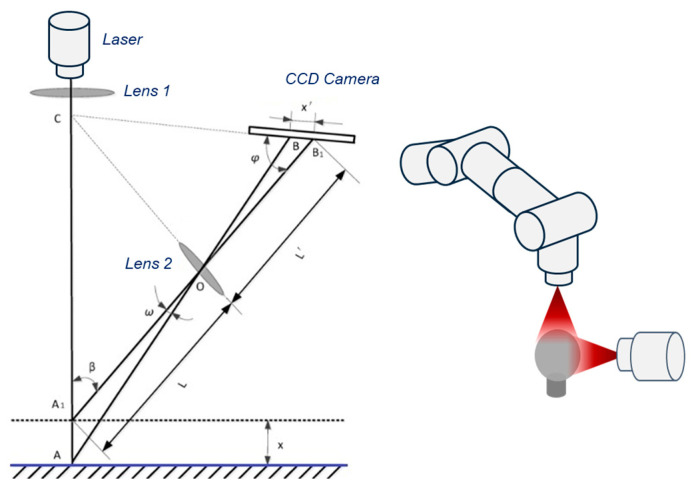
The scheme of laser triangulation based on [27,28] and the position of the probe head during measurements.

**Figure 5:**
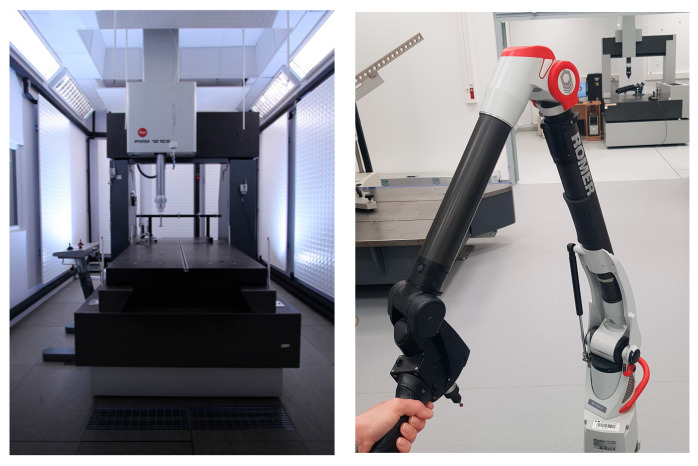
Leitz PMM 12106 Coordinate Measuring Machine, Romer Absolute Arm 7320RI with Scanner RS2 laser head.

The main measurement procedure included ten scans of the surfaces of six spheres, presented in Figure 2, carried out in two stages: before and after matting. The Absolute Arm 7320RI measuring system with a Scanner RS2 laser head and Polyworks suite 2023 software from InnovMetric Software Inc., Quebec, QC, Canada were used in the tests. The laser head operates based on the principle of laser triangulation, the schematic of which is presented in Figure 4. Photographs of the mentioned equipment used are shown in Figure 5. During the emission of the laser beam, point A is formed on the surface of the object, while a corresponding point B appears on the surface of the CCD camera. A shift in the position of point A to A1 by a value of x on the CCD camera by x′ [27,28]. The triangulation method utilizes the similarity of triangles, making use of trigonometric properties, as shown in Equation (1):

(1) $$x=\frac{Lx^{\prime }sin\varphi }{L^{\prime }sin\beta -x^{\prime }sin(\varphi +\beta )}$$

where the components of the equation are as follows:

x—Displacement of the point on the measured object;

x′—Displacement of the corresponding image point on the CCD camera;

β—Angle between the initial laser beam and the axis of Lens 2;

φ—Angle between the CCD camera and the axis of Lens 2;

L—Distance from the measured object (point A) to Lens 2;

L′—Distance from the image point to Lens 2.

The maximum permissible error (MPE) equation describing a Scanner RS2 laser head is equal to 0.065 mm. The MPE was established based on ISO 10360-8:2013 [29]. For each variant (with and without matting), each sphere was measured in two cycles, each consisting of ten individual scans. The first ten scans were performed from the top position, while the next ten were taken from the side (at 90°) (Figure 4). The measurement procedure in metrological applications is preceded by the mandatory calibration of the machine. In the case of coordinate measuring machines (CMMs), calibration is performed using a reference sphere with known dimensions and a valid calibration certificate and generally is performed automatically. This step is essential to ensure measurement consistency.

Points from reflections were removed from the point cloud by indicating the area to be removed. Spheres were then created using the best-fit method—the least squares method applied to the obtained points, with a 3-sigma filter. The diameters were determined from the filtered point data. The uncertainties were determined using the multi-position method in accordance with ISO 15530-2 (draft) [30] and an internal procedure, with the use of a golden sphere with a corresponding diameter. The multi-position method assumes repeating the measurement in different locations and settings. The uncertainty budget consists of component uncertainties including related to repeatability, machine geometric errors, standard measurement, and standard and environment temperature. The results obtained from the tests were compared with reference data obtained from the sphere calibration process on the Leitz PMM 12106 coordinate measuring machine. The maximum permissible error (MPE) equation describing a PMM machine is MPE = 0.0008 + 0.0025/1000 L mm (Figure 5). The uncertainty of determining nominal sphere diameters according to the method of least squares is 0.12 µm, based on [30].

**Figure 6:**
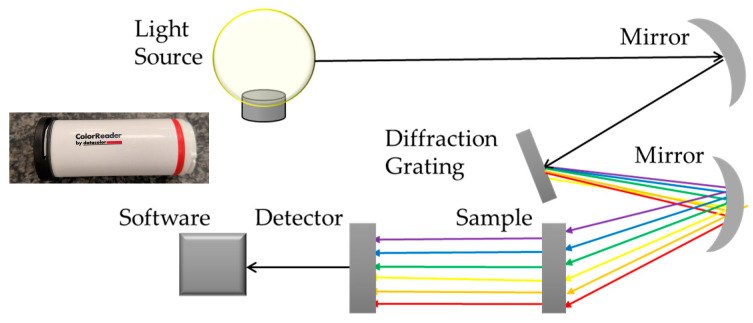
Datacolor ColorReader and the principle of spectrocolorimeter operation.

This research was supplemented with color measurements of the measured spheres before and after the matting process. This was to demonstrate that despite the spheres being covered with a matting product, the original surface still ‘breaks through’ the matting layer to some extent. The measuring device used was a ColorReader from Datacolor (Lucerne, Switzerland) (Figure 6), equipped with an advanced optical lens and six white LEDs with a high Color Rendering Index (CRI), which isolates and evenly illuminates colors. The CRI is a key lighting parameter and determines the correctness of perceived colors. The principle of spectrocolorimeter operation is presented in Figure 6.

## 3. Results

### 3.1. Results of Determining the Diameters of Reference Spheres from Various Materials

#### 3.1.1. Preliminary Tests

**Figure 7:**
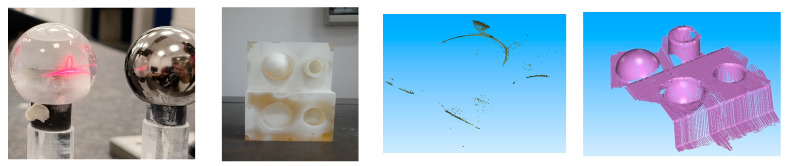
From left to right: laser penetration effect, matted standard for optical measurements, point cloud acquired from the unmatted standard, and point cloud acquired from the matted standard.

Figure 7 shows the effect of a light beam penetrating the glass surface of a sphere. Measuring such an object without prior whitening is completely impossible. The laser penetrates the material, causing the point cloud to split and the points to overlap, which makes accurate measurement impossible.

In the next step, the laser’s performance was evaluated on a semi-transparent standard. For this purpose, the laser beam was directed onto the object’s surface. It was observed that acquiring a point cloud by scanning this type of surface without prior matting was impossible (point cloud shown on the left). Only after the application of the Aesub Blue matting spray was it possible to capture the point cloud (right), thereby enabling accurate surface reconstruction (Figure 7).

#### 3.1.2. Results of the Diameters of Reference Spheres Measured Without Matting

**Figure 8:**
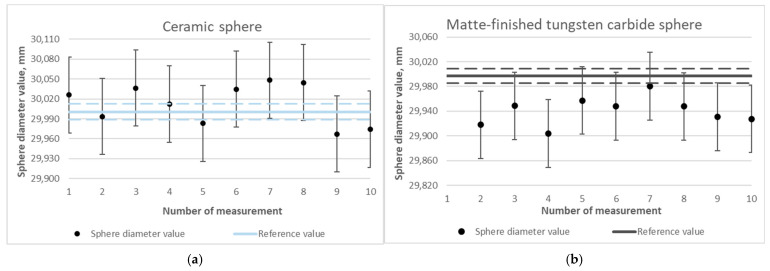

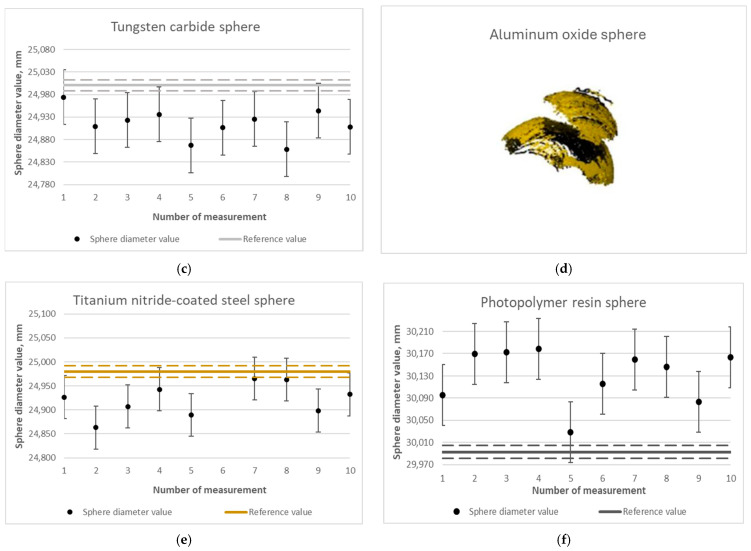
Determined values of the diameters of spheres made of selected materials using optical measurement without the matting process: (**a**) ceramic sphere, (**b**) matte-finished tungsten carbide sphere, (**c**) tungsten carbide sphere, (**d**) aluminum oxide sphere, (**e**) titanium nitride-coated steel sphere, and (**f**) photopolymer resin sphere.

The measurement results of spheres made of various materials were analyzed by comparing the obtained diameter values to the reference values, including the uncertainty in determining the reference values (dashed lines). The results are presented in graphs (Figure 8) and in Table 1.

The lowest difference of 0.012 mm was obtained when measuring a ceramic sphere (Figure 8a). Comparable differences, which equal approximately 0.06 mm compared to the reference value, were observed for the spheres made of matte-finished tungsten carbide (Figure 8b) and steel coated with titanium nitride (Figure 8e). For a tungsten carbide sphere, a highly reflective one, the obtained difference value is at the level of 0.085 mm (Figure 8c), which confirms the concept that the largest errors can be expected in highly reflective materials. An unfavorable effect of the reflection of the laser beam was observed in the measurement of the aluminum oxide sphere (Figure 8d), resulting in a strong scattering of the acquired point cloud. The measurement was discontinued without matting for this sphere. The result for photopolymer resin of 0.138 mm (Figure 8f) is higher in comparison to the reference value from tactile measurement. This situation confirms the validity of the use of matting sprays.

Due to their high accuracy, tactile (contact) measurement results are used in this study as nominal reference values. The deviations observed between optical and contact measurement results vary depending on the material of the measured spheres. These differences confirm the sensitivity of optical measurement systems to the factors discussed in the introduction, including those related to surface properties—such as the material’s refractive index.

#### 3.1.3. Results of the Diameters of Reference Spheres Measured with Matting

**Figure 9:**
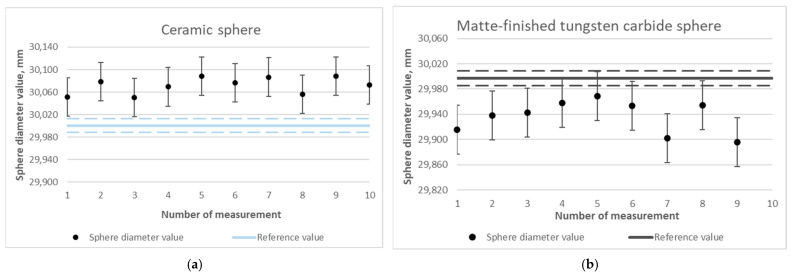

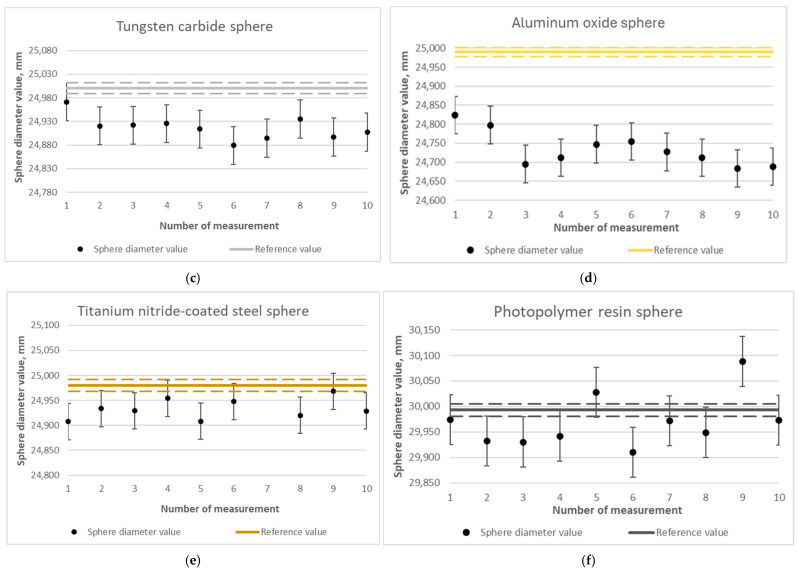
Determined values of the diameters of spheres made of selected materials using optical measurement with the matting process: (**a**) ceramic sphere, (**b**) matte-finished tungsten carbide sphere, (**c**) tungsten carbide sphere, (**d**) aluminum oxide sphere, (**e**) titanium nitride-coated steel sphere, and (**f**) photopolymer resin sphere.

The next stage of work involved repeating the test from Section 3.1.2, this time after applying a matting layer on the surfaces of the spheres. The results are presented in graphs (Figure 9) and Table 2.

The result for measuring the diameter of the ceramic sphere was 0.072 mm (Figure 9a), differing more from the measurement value before matting, which amounted to 0.012 mm (Figure 8a); matting resulted in an excess of 0.06 mm—which is justified and is within the limits declared by the manufacturer of the matting products. Comparable deviations were received for spheres made of matte-finished tungsten carbide (−0.061 mm, Figure 9b) and steel coated with titanium nitride (−0.047 mm) (Figure 9e). For the highly reflective tungsten carbide sphere, the observed deviation was −0.083 mm (Figure 9c). This provides an interesting result in the context of incomplete coverage with the matting layer. In the case of the aluminum oxide sphere (Figure 9d), the measurement was possible and resulted in a deviation of −0.256 mm, which still represents a significant discrepancy compared to the other results. However, the key point is that the matting process enabled point acquisition in that case. The result for the photopolymer resin sphere is now highly satisfactory, with a deviation of only −0.023 mm (Figure 9f).

**Figure 10:**
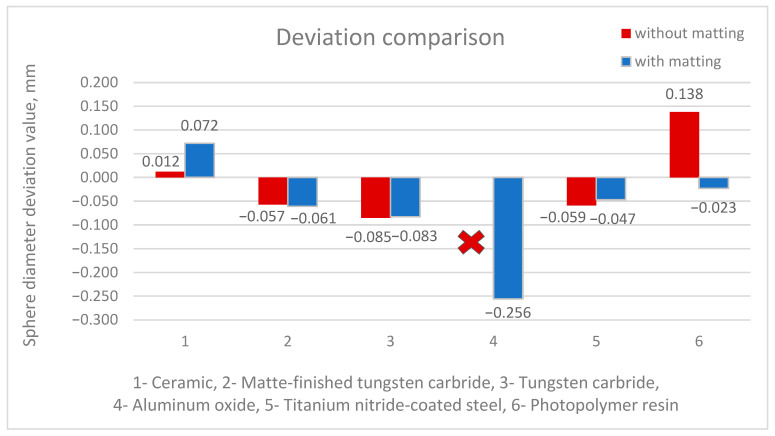
Comparison of deviations of diameter values of spheres made of selected materials without and with matting process.

A comparison of the deviations is shown in Figure 10. The results of matte-finished tungsten carbide and steel with a titanium nitride coating are at a similar level to those obtained without matting, approximately −0.05 mm (Figure 10), with a more favorable trend observed for the titanium nitride-coated steel. The measurement results of a tungsten carbide sphere—a highly reflective material—were similar to those obtained without spray. A larger deviation compared to the other materials (−0.084 mm) was observed, indicating that matting the surface did not significantly improve the results in this case. However, a clear beneficial effect of matting was observed for both

− The photopolymer resin sphere, for which the diameter value was definitely closer to the reference one (0.138 mm—deviation without matting, −0.023 mm—deviation with matting),
− The aluminum oxide sphere, where matting enabled the acquisition of measurement data; nevertheless, they still differ significantly from the reference values (−0.256 mm—deviation with matting).

**Figure 11:**
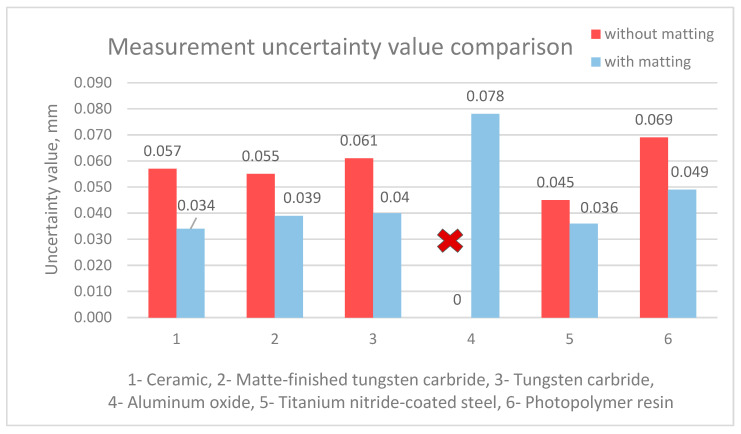
Comparison of uncertainty values of sphere measurements made of selected materials without and with matting process.

In summary, the use of the matting process proved essential for the aluminum oxide sphere, as it did for the photopolymer resin sphere. For the other spheres, the differences in the measured diameters were not as significant; however, a key finding is that the matting process substantially reduced the measurement uncertainty (Figure 11). Therefore, the authors recommend the use of matting sprays. In cases where the benefit of using a matting product is uncertain, it is advisable to monitor the measurement process—an experienced operator can often detect potential issues during scanning by observing the point acquisition rate and the spatial distribution of the collected points.

### 3.2. Results of Color Tests—Supplementary

Based on the results obtained in Section 3.1, the authors decided to supplement the research with an examination of the matting process—specifically, its effectiveness, which was assessed repeatedly measuring the color of the spheres before and after the application of the matting layer using a spectrocolorimeter. The results are presented in Table 3. Difficulties were encountered when measuring a highly reflective tungsten carbide sphere. The results confirmed the thesis that the original color of the measured object ‘breaks through’ the matting layer. The matting did not result in completely similar color coatings. This is especially important when using self-sublimating sprays, where over time the thickness of the matting layer decreases, revealing the original surface. This may significantly affect the quality of the measurement.

## 4. Discussion

Based on the observed regularities, practical experience, and scientific sources, the authors propose a conceptual classification of objects into two groups, easy to scan and hard to scan (Table 4), highlighting the most commonly encountered situations. The table is intended for illustrative purposes; the criteria may vary slightly depending on the measurement system used.

Primarily for materials classified as hard-to-scan, the authors propose introducing certain improvement activities at all stages of the measurement process:

I. Pre-measurement stage

− Matting the surface of the object being measured by applying the powder.
  (In the case of objects with more complex geometries or surface finishes, the authors recommend the use of a self-sublimating spray, as manual removal of permanent spray may be problematic in hard-to-reach areas. However, the sublimation time should be considered, and the product should be selected accordingly to match the anticipated measurement duration.)
− Etching the surface layer of the measured object (less common).

II. Measurement stage

− Optimal selection of scanning parameters, including the problematic type of the surface: number of points, resolution, beam and intensity.

III. Post-measurement stage

− Point cloud filtration, e.g., using the least squares method, noise reduction, or model polygonization.
− Manual reduction of the point cloud with elements originating from reflections.

Nevertheless, it must be acknowledged that, despite the reasonable implementation of the aforementioned processes, attaining a level of accuracy comparable to that of easy-to-scan objects remains challenging in the case of hard-to-scan objects.

The presented measurement results highlight the significant influence of the material of the measured object and the condition of its surface (in this case, with or without matting) on the accuracy of coordinate optical measurements. It was observed that the matting process, in some cases, significantly improved the measurability of the objects, and even made scanning possible. An interesting finding is also the expanded measurement uncertainties calculated for the analyzed objects before and after matting. The uncertainties for matted objects were, on average, 30% lower than those for the non-matted objects, indicating greater repeatability of results when matting is applied.

In the context of the referenced studies, in which the authors emphasize the dual impact of transparent materials, causing both a shift in measurement values and an increase in uncertainty due to the varying quality of surface reconstruction [12], and study [13], which identifies a systematic noise error dependent on the object’s color, the authors, being aware that properties such as transparency and color may lead to varying levels of measurement distortion, undertook an investigation to determine whether matting fully covers the measured surface and homogenizes its color. An important finding was the incomplete surface coverage by the matting agent, which may be particularly critical when using a reference artifact made from a hard-to-scan material. This is because the inherent properties of the material may still affect measurement accuracy despite the applied coating. This effect is especially pronounced in the case of self-sublimating sprays, where the influence of the original surface increases over time. For such cases, the authors anticipate higher scan accuracy in the initial moments of scanning, followed by a gradual decline.

## 5. Conclusions

The conducted research and analyses confirm the versatility and practical applicability of coordinate optical measurement systems. The authors draw attention to the high sensitivity of such systems to environmental influences and to the material and geometrical characteristics of the measured object, emphasizing the distinction between easy-to-scan and hard-to-scan surfaces. The latter category includes surfaces that are shiny, transparent, translucent, varnished, black, or structurally complex. For hard-to-scan objects, the authors propose several approaches presented in the study, including the application of matting products (demonstrated to reduce expanded measurement uncertainty by approximately 30%), the optimal selection of scanning parameters, data filtering, and point cloud reduction. These methods offer clear advantages, including increased measurement accuracy, and in some cases, enable measurements that would otherwise be unfeasible. Nonetheless, it must be acknowledged that interventions affecting the measured surface may introduce undesirable effects. For example, matting may not provide complete and uniform coverage, potentially affecting data integrity; similarly, etching may result in material loss, and excessive data filtering may lead to a loss of critical information. These limitations must be considered by users of optical systems. Furthermore, the proposed procedures typically require additional processing time (involving both the operator and the measurement system) and may incur additional costs, particularly in relation to effective surface matting. For this reason, the authors highlight the importance of selecting an optical measurement system appropriately matched to the specific application and measurement conditions. This study also underscores the value of multisensory systems that integrate contact-based measurements, which may be advantageous for objects that are particularly difficult to scan using optical methods alone. Although contact measurements are generally more time consuming, in cases involving complex or problematic materials, they may prove to be a more accurate and economically viable solution.

## Figures and Tables

**Table 1:** Summary of the results of the average values of the diameters of spheres made of selected materials during optical measurement without the matting process.

Sphere		Nominal Value, mm	Measured Value, mm	Uncertainty, mm	Difference, mm
(a) Ceramic (AQ662)	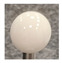	30.00050	30.012	0.057	0.012
(b) Matte-finished tungsten carbide (136091)	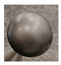	29.99723	29.940	0.055	−0.057
(c) Tungsten carbide (137518)	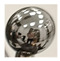	25.00039	24.915	0.061	−0.085
(d) Aluminum oxide (D0953)	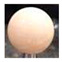	24.99013	not-measured	-	-
(e) Titanium nitride-coated steel (LMW_OPT_25)	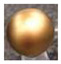	24.98043	24.921	0.045	−0.059
(f) Photopolymer resin	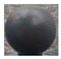	29.99300	30.131	0.069	0.138

**Table 2:** Summary of the results of the average values of the diameters of spheres made of selected materials during optical measurement with the matting process.

Sphere		Nominal Value, mm	Measured Value, mm	Uncertainty, mm	Difference, mm
(a) Ceramic (AQ662)	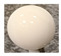	30.00050	30.072	0.034	0.072
(b) Matte-finished tungsten carbide (136091)	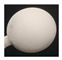	29.99723	29.936	0.039	−0.061
(c) Tungsten carbide (137518)	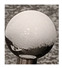	25.00039	24.917	0.040	−0.083
(d) Aluminum oxide (D0953)	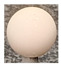	24.99013	24.734	0.078	−0.256
(e) Titanium nitride-coated steel (LMW_OPT_25)	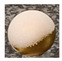	24.98043	24.933	0.036	−0.047
(f) Photopolymer resin	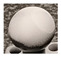	29.99300	29.970	0.049	−0.023

**Table 3:** Results of measuring the color of the tested spheres before and after the matting process.

Sphere	Code	Without Matting	Code	After Matting
Ceramic (AQ662)	#e6dfd3		#e5e4d8	
Matte-finished tungsten carbide (136091)	#7a6e7a		#cfd1d2	
Tungsten carbide (137518)	#8a8453		#a2a1ad	
Aluminum oxide (D0953)	#dacbb2		#e3d8cf	
Titanium nitride-coated steel (LMW_OPT_25)	#dda959		#cbb79d	
Photopolymer resin	#525762		#b3b8bc	

**Table 4:** Division of objects due to the ease of the scanning process.

Parameter	Easy-to-Scan		Hard-to-Scan	
Material properties	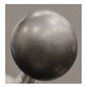	Matte Gloss Range 0–10 GU * Reflectance Range 0–20%	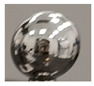	Glossy, Transparent, Translucent, Varnished Gloss Range 70–100 GU * Reflectance Range 60–100%
Color	Light color L (50–100) **		Black color L (0–50) ** also red, green, depending on the system used	
	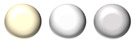		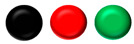	
Form	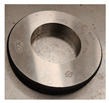	Simple free access to measured surfaces, e.g., basic geometric element	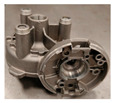	Complex, difficult access to measured surfaces, e.g., deep holes

* Gloss Units measured typically at a 60° angle, according to ISO 2813 [31]. ** CIELAB Color Model (L*a*b*) L* = Lightness, ranges from 0 to 100, where: 0 = black (darkest), 100 = white (lightest).

## Data Availability

The original data presented in the study are openly available in RODBUK at https://doi.org/10.58099/PK/IP2A6X.
